# Black Pepper and Dietary Lipids Synergistically Enhance Carotenoid Bioavailability: A Narrative Review

**DOI:** 10.1093/nutrit/nuaf248

**Published:** 2026-05-26

**Authors:** Minna Luo, Hang Xiao

**Affiliations:** Department of Food Science, Institute for Applied Life Sciences, University of Massachusetts, Amherst, MA, 01003, United States; Department of Food Science, Institute for Applied Life Sciences, University of Massachusetts, Amherst, MA, 01003, United States; Department of Microbiology, Institute for Applied Life Sciences, University of Massachusetts, Amherst, MA, 01003, United States

**Keywords:** carotenoids, human trials, black pepper, dietary lipids, bioavailability, piperine, emulsions

## Abstract

Carotenoids are lipophilic micronutrients with significant health benefits, yet their poor bioaccessibility and bioavailability limit their physiological impact. Evidence suggests that co-ingestion of black pepper (piperine) and dietary lipids can synergistically enhance carotenoid utilization. This narrative review evaluates the scientific basis for this interaction. A randomized crossover human clinical trial clearly demonstrated that black pepper and a canola oil emulsion together markedly enhanced carotenoid absorption, and in vitro studies provide evidence for the ability of lipids and piperine to improve carotenoid uptake. Mechanistically, dietary lipids primarily act at the bioaccessibility level by promoting micellization, while piperine influences both bioaccessibility (eg, by altering micelle dynamics and intestinal permeability) and bioavailability (eg, by inhibiting efflux transporters and metabolic enzymes). These complementary actions produce a synergistic enhancement of carotenoid utilization. Despite promising findings, further studies are needed to clarify the mechanisms, establish dose–response relationships, and assess long-term efficacy and safety. Overall, combining black pepper and dietary lipids represents a practical, culturally relevant, and cost-effective dietary strategy for improving carotenoid bioavailability, with potential implications for preventing vitamin A deficiency and reducing chronic disease risk.

## INTRODUCTION

Carotenoids are a diverse class of lipophilic pigments, including β-carotene, lutein, lycopene, and α-carotene, and are found abundantly in fruits and vegetables.^1^ These compounds are associated with a wide array of health benefits, such as antioxidant protection, immune modulation, and reduced risk of chronic diseases, including cardiovascular disease and certain cancers.^2^^,^^3^ However, the bioefficacy of carotenoids is constrained, not solely by dietary intake but more critically by their bioaccessibility and bioavailability. Bioaccessibility refers to release, solubilization into micelles, and availability for absorption. Bioavailability refers to the fraction that actually enters the systemic circulation and becomes physiologically available.

Despite their abundance in plant-based foods, carotenoid bioavailability is often low and highly variable, influenced by food matrix composition, processing methods, co-ingested dietary factors, and host physiological traits.^4^ Previous reviews have explored these factors individually, particularly the role of dietary lipids in enhancing carotenoid absorption through micellar solubilization.^5^ More recent work has examined bioenhancers such as piperine, an active compound in black pepper, known for its capacity to inhibit efflux transporters and metabolic enzymes, thereby enhancing nutrient absorption.^6^^,^^7^ However, the synergistic effects of and underlying mechanisms for piperine and dietary lipids in enhancing carotenoid bioavailability have been less thoroughly reviewed.

This narrative review addresses this gap by critically examining the scientific rationale and emerging evidence for the synergistic enhancement of carotenoid bioavailability through the co-consumption of black pepper and dietary lipids. It evaluates findings from a randomized human clinical trial and in vitro studies, dissects mechanistic pathways involved in absorption and metabolism, and discusses implications for dietary guidance and functional food design. By focusing on this underexplored but promising interaction, this review aims to provide a novel and translational perspective on improving carotenoid utilization through common, culturally acceptable dietary strategies.

## EFFECTS OF DIETARY LIPIDS AND PIPERINE ON CAROTENOID BIOAVAILABILITY

**Figure 1.**
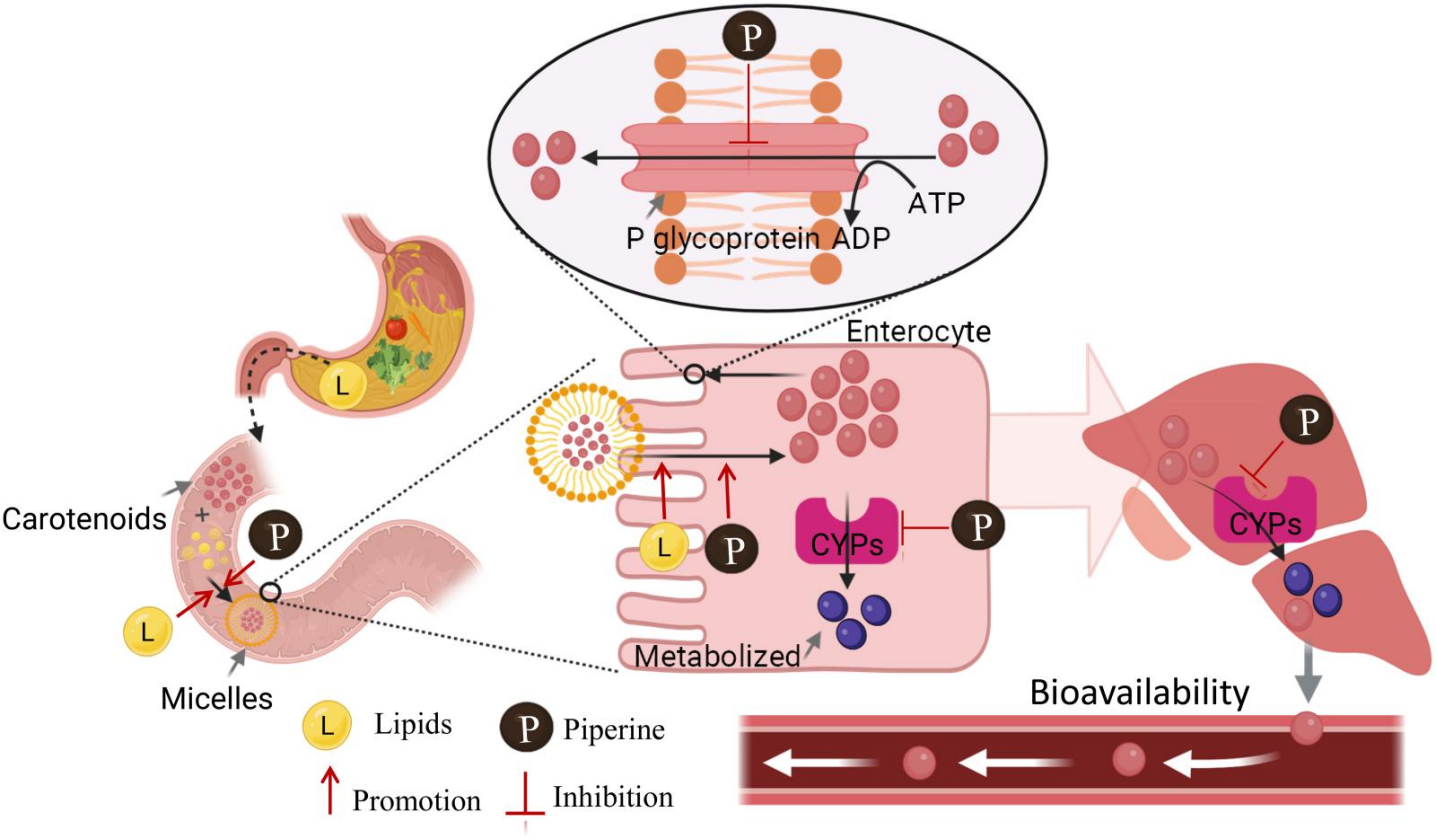
Mechanisms of Carotenoid Absorption and the Role of Dietary Lipids and Piperine in Enhancing Bioavailability

The absorption of carotenoids from ingestion to systemic circulation involves multiple steps, each of which can significantly affect their overall bioavailability (as illustrated in Figure 1). Numerous factors influence this process, including the food matrix, processing methods, co-consumed dietary factors, and individual physiological conditions, all of which contribute to the overall bioavailability of carotenoids in humans.^8^ Among these factors, dietary lipids and piperine have received particular attention for their potential to enhance carotenoid bioavailability by modulating digestive and metabolic processes.

### The Journey from Food to Systemic Circulation

Carotenoids in plant foods are often located within cellular structures such as chloroplasts and chromoplasts, or may exist in crystalline form, making them relatively inaccessible.^4^ The initial and crucial step for absorption is their release from this food matrix. In the oral cavity, mastication reduces food particle size, while salivary mucins dilute and destabilize the food matrix, thereby facilitating the subsequent release of carotenoids during gastric and intestinal digestion. Moreover, salivary amylase and lingual lipase act mainly on starch and triglycerides, which can further release carotenoid from the food matrix. The subsequent digestive processes, including the action of gastric acid and enzymes like pepsin (a protease) and gastric lipase, break down the cellular structures of the food. Once released, carotenoids, being lipophilic, must be incorporated into mixed micelles to traverse the aqueous environment of the small intestine and reach the absorptive surface of enterocytes. This micellarization process is heavily reliant on dietary fat digestion.^4^ In the duodenum, dietary triglycerides are hydrolyzed by pancreatic lipase and colipase into 2-monoacylglycerols and free fatty acids. These lipolytic products, along with bile salts secreted by the gallbladder (stimulated by cholecystokinin release in response to dietary fat), phospholipids, and cholesterol, aggregate to form mixed micelles. Carotenoids partition into the hydrophobic core of these micelles, effectively solubilizing them in the gut lumen. The efficiency of micellarization is a key rate-limiting step for carotenoid absorption. The carotenoid-loaded micelles diffuse to the brush border membrane of enterocytes. Carotenoid uptake into enterocytes is thought to occur via a combination of passive diffusion and carrier-mediated transport.^9^ Inside the enterocytes, carotenoids are metabolized by cytochrome P450 enzymes, and actively effluxed by transporters like P-glycoprotein, further diminishing carotenoid availability.^10^^,^^11^ Unchanged carotenoids and metabolites are subsequently incorporated into chylomicrons, which are large lipoprotein particles assembled in the endoplasmic reticulum.^12^ Chylomicrons are secreted from the basolateral membrane of enterocytes into the lymphatic system, bypassing the portal circulation to the liver initially.^12^ They enter the systemic bloodstream via the thoracic duct. In circulation, lipoprotein lipase (LPL), an enzyme located on the endothelial surface of capillaries in muscle and adipose tissue, hydrolyzes the triglycerides within chylomicrons, releasing fatty acids for tissue uptake or energy. This process results in the formation of chylomicron remnants, which are relatively enriched in carotenoids and cholesterol esters. These remnants are subsequently taken up by the liver through receptor-mediated endocytosis. In the liver, carotenoids can be stored, metabolized further, or re-secreted into circulation, packaged within other lipoproteins, such as very-low-density lipoproteins (VLDLs), low-density lipoproteins (LDLs), and high-density lipoproteins (HDLs), for delivery to peripheral tissues.^13^

### Role of Dietary Lipids in Carotenoid Bioaccessibility

Dietary lipids are indispensable for the efficient absorption of carotenoids.^5^ Luo et al demonstrated in a randomized crossover clinical trial that adding a canola oil emulsion to a carotenoid-rich vegetable salad significantly increased plasma carotenoid concentrations.^14^ The mechanisms involve micelle formation, lipid composition, and cellular transport. First, dietary lipids facilitate micelle formation, thereby enhancing the bioaccessibility of carotenoids. As lipophilic compounds, carotenoids must be incorporated into lipid droplets during digestion to be effectively absorbed. Tan et al demonstrated that increasing corn oil concentrations from 2.5% to 10% significantly improved β-carotene bioaccessibility.^15^ Yao et al (2021) reported that increasing lipid levels in nanoemulsions significantly improved the intestinal absorption of carotenoids in a mouse model.^16^ These results highlight the critical role of lipids in promoting carotenoid micellization. Second, the type of dietary lipids significantly impacts absorption efficiency. A recent human clinical study compared 5 oils—olive oil and canola oil (both mono-unsaturated fatty acid [MUFA]-rich), and corn, sunflower, and flaxseed oils (polyunsaturated fatty acid [PUFA]-rich)—and demonstrated that MUFA-rich oils significantly enhanced carotenoid absorption compared with PUFA-rich oils.^14^ This result indicated that the saturation level of dietary lipids modulates carotenoid bioaccessibility. Moreover, the carbon chain length of dietary lipids also impacts carotenoid absorption. Long-chain triglycerides (LCTs) are more effective than medium-chain triglycerides (MCTs) in enhancing carotenoid bioaccessibility.^17^ Lastly, dietary lipids can modulate the expression of intestinal transporters.^18^ For instance, the presence of dietary oil, such as linoleic acid, has been shown to upregulate PPARγ and SR-B1 expression in rat intestines, thereby activating lipid transport pathways and enhancing carotenoid absorption.^19^

### Mechanisms for the Role of Piperine in Carotenoid Bioaccessibility and Bioavailability

Piperine, the primary active alkaloid in black pepper (*Piper nigrum*), is renowned for its ability to enhance the bioaccessibility and bioavailability of a wide range of structurally and functionally diverse compounds, including pharmaceuticals, phytochemicals, and micronutrients. It can significantly enhance the bioavailability of carotenoids in both in vitro models^20^ and in human studies.^21^^,^^22^ This bioenhancing effect is attributed to several mechanisms of action.

#### Inhibition of Metabolic Enzymes

Piperine is known to inhibit drug-metabolizing enzymes, particularly those involved in first-pass metabolism in the liver and intestines. It has been shown to block the activity of cytochrome P450 enzymes, especially CYP3A4.^10^^,^^23^^,^^24^ Since CYP3A4 is involved in the oxidative metabolism of many compounds, including possibly some carotenoids, its inhibition by piperine can slow down carotenoid breakdown and allow more of the active compound to be absorbed into the circulation.

#### Inhibition of Efflux Transporters

Another important mechanism involves piperine’s inhibition of efflux transporters, particularly P-glycoprotein, an ATP-dependent pump located on the apical membrane of intestinal epithelial cells. P-glycoprotein actively transports absorbed compounds back into the intestinal lumen, thereby reducing their net absorption. By inhibiting P-glycoprotein activity, piperine decreases the efflux of carotenoids, leading to greater intracellular retention and enhanced transfer into the systemic circulation.^25^^,^^26^

#### Enhanced Intestinal Permeability

Piperine may also enhance carotenoid absorption by increasing intestinal membrane permeability. Due to its lipophilic nature, piperine can interact with phospholipid bilayers, altering membrane dynamics and fluidity. This interaction may promote the passive diffusion of carotenoids across enterocytes. It has been hypothesized that piperine may form nonpolar complexes with nutrients, further facilitating their transcellular transport.^27^ In addition, piperine has been reported to stimulate gastric acid secretion and modulate tight junction integrity, both of which could contribute to enhanced nutrient uptake.^28^

#### Promotion of Micelle Formation

Efficient micellar solubilization is critical for the intestinal absorption of lipophilic nutrients such as carotenoids. Piperine may enhance micelle formation indirectly by stimulating bile acid secretion and inhibiting bile acid metabolism.^29^^,^^30^ This results in increased bile salt concentrations in the intestinal lumen, promoting the solubilization of carotenoids into mixed micelles and thereby improving their bioaccessibility.

## SYNERGISTIC ENHANCEMENT OF CAROTENOID BIOAVAILABILITY BY BLACK PEPPER/PIPERINE AND DIETARY LIPIDS

**Figure 2.**
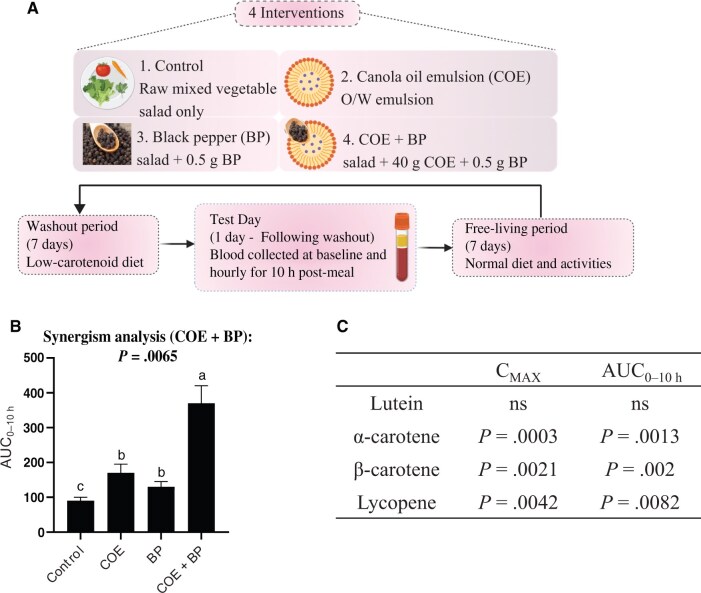
(A) The study design of the randomized crossover human trial. (B) Area Under the Curve (AUC_0–10 h_, nmol/L*10 h) of total carotenoids in the plasma of human subjects during the first 10 h after consumption of 4 different test meals. (C) Synergism analysis for C_MAX_ and AUC_0–10 h_ of lutein, α-carotene, β-carotene, and lycopene. Synergism analysis was performed based on the method used in previous studies.^31^ Data represents mean ± standard error. Different superscript letters represent statistical differences between the various groups (ANOVA, *P* < 0.05, *n* = 16). O/W emulsion: oil in water emulsion. Redrawn, but based on Luo et al 2022, with permission from Elsevier.^21^

The individual roles of dietary lipids in facilitating carotenoid solubilization and micellar transport, and of piperine in modulating gut physiology and metabolism, are well recognized. The central hypothesis of this review is that these 2 factors act synergistically to enhance carotenoid bioavailability to a greater extent than either component alone. Evidence supporting this synergy comes from a combination of a human clinical trial and in vitro studies. A notable human clinical study conducted by Luo et al (2022) demonstrated a significant synergistic effect between black pepper (piperine) and dietary lipids on carotenoid bioavailability.^21^ This study investigated the synergistic effects of black pepper and a canola oil-based emulsion (COE) on the bioavailability of carotenoids from raw mixed vegetables in healthy young adults. The research employed a randomized crossover design where 16 participants (aged 28 ± 2 years, BMI 22.6 ± 0.4 kg/m^2^) consumed 4 different test meals after a 7-day washout period: (1) a raw vegetable salad (control), (2) salad with 0.5 g of black pepper, (3) salad with 40 g of COE (containing 8 g of canola oil), and (4) salad with both COE and black pepper (COE + black pepper). Blood samples were collected over 10 hours post-meal consumption, and the triacylglycerol-rich lipoprotein (TRL) fraction, representing newly absorbed carotenoids, was analyzed for lutein, α-carotene, β-carotene, and lycopene levels. Key pharmacokinetic parameters such as the maximum plasma concentration (Cmax) and the area under the curve from 0 to 10 hours (AUC_0 − 10 h_) were determined for total and individual carotenoids (Figure 2A). Synergistic analysis was conducted by summing the mean values and variances of the individual effects of black pepper and the emulsion, followed by calculating the corresponding SDs. A 2-way analysis of variance was then used to assess statistical significance.^31^

The main results demonstrated that the combination of COE and black pepper significantly enhanced carotenoid bioavailability more effectively than either black pepper or COE alone. Specifically, the COE + black pepper group showed a 6.1-fold increase in the AUC_0 − 10 h_ of total plasma carotenoids compared with the control group, a 2.1-fold increase compared with the black pepper group, and a 3.0-fold increase compared with the COE group. For individual carotenoids, COE + black pepper increased the AUC_0 − 10 h_ of plasma lutein, α-carotene, β-carotene, and lycopene by 4.8, 9.7, 7.6, and 5.5-fold, respectively, compared with the control. A significant synergistic interaction between COE and black pepper was observed in increasing both C_max_ and AUC_0 − 10 h_ for total carotenoids, α-carotene, β-carotene, and lycopene (Figure 2B and 2C). In sum, this human study demonstrated that combining black pepper with a COE synergistically enhances the bioavailability of various carotenoids from raw vegetables. Ex vivo studies also demonstrated that piperine increased β-carotene uptake by intestinal segments by as much as 2.5-fold.^7^

While existing evidence, particularly from the study by Luo et al (2022),^5^ is compelling, the underlying mechanisms driving the synergistic effects of dietary lipids and black pepper remain unclear. Future research should aim to elucidate these mechanisms. In addition, well-designed human clinical trials are needed to systematically assess this 3-way interaction across a wider range of dietary carotenoids, diverse food matrices, and defined doses of both lipids and piperine.

## MECHANISMS UNDERPINNING SYNERGISTIC EFFECTS OF DIETARY LIPIDS AND PIPERINE

The synergy likely arises because lipids and piperine address different, yet complementary, bottlenecks in the carotenoid absorption pathway. Lipids are crucial for the initial stages of carotenoid absorption, by solubilizing them in micelles and transporting them to the enterocyte surface.^5^ Piperine then acts at the enterocyte level and beyond, enhancing membrane permeability, inhibiting efflux pumps that would otherwise expel carotenoids, and reducing metabolic degradation both intestinally and systemically.^32^ This combined action can lead to a multiplicative, rather than merely additive, improvement in bioavailability.

### Lipids Create a Favorable Environment for Piperine’s Action on Carotenoids

The synergistic enhancement of carotenoid absorption by piperine and dietary lipids is mediated by their shared lipophilicity and co-localization within lipid digestion products. Dietary lipids facilitate the emulsification and micellar solubilization of carotenoids, enabling absorption at the enterocyte border.^4^ Piperine, a lipophilic compound,^33^ is likely co-solubilized into the same lipid droplets and mixed micelles. This co-localization enhances the absorption of piperine and positions it at the absorption site, where it can modulate membrane dynamics and inhibit enzymes and transporters, thereby improving carotenoid bioavailability.

### Piperine Affects the Intestinal Lipid Microenvironment

Piperine can alter the lipid dynamics of the intestinal cell membrane. Due to its lipophilic nature, piperine can interact with phospholipid bilayers, altering membrane dynamics and fluidity.^27^ This suggests that piperine may integrate into the enterocyte’s brush border membrane, interacting with phospholipids and cholesterol. Such interactions could lead to an increase in membrane fluidity and/or the creation of transient pores.

Dietary lipids are crucial in forming micelles that transport carotenoids to this piperine-modified membrane.^16^^,^^34^ The increased fluidity or altered structure of the membrane then facilitates more efficient partitioning of carotenoids within the membrane surface and promotes easier diffusion or transport of carotenoids from the micelle into the enterocyte. This direct interaction at the membrane interface, where lipids deliver the cargo and piperine “opens the gate,” could be a core element of the synergy.

### Combined Effects on Micellarization and Carotenoid Stability

While dietary fats mainly drive micelle formation, piperine may also play a role. Piperine can be incorporated into micelles with carotenoids and might affect their size, stability, or ability to carry carotenoids, though this idea is still speculative and needs more research. Additionally, carotenoids are prone to breakdown in the gut, due to acidic and oxidative conditions.^35^ Since piperine has antioxidant properties,^6^ it may help protect carotenoids from degradation before absorption, increasing the quantity of intact carotenoids that reach the intestinal lining.

### Modulation of Metabolism or Transporters by Piperine in a Lipid-Rich Context

Piperine may enhance carotenoid levels by inhibiting enzymes that normally break them down. These include cytochrome P450s and phase II enzymes such as CYP3A4 and UGTs, which metabolize carotenoids in the intestine and liver through processes such as oxidation and conjugation.^10^^,^^23^^,^^24^ By blocking these enzymes, piperine reduces carotenoid degradation, allowing more to enter the bloodstream and reach the tissues. Since carotenoids pass through the liver after absorption, piperine offers “double protection” by slowing their breakdown in both the gut and liver. Lipids help with initial absorption, while piperine supports greater retention after liver processing. Moreover, piperine is known to inhibit P-glycoprotein, an efflux transporter that can pump compounds, including carotenoids or their metabolites, back into the intestinal lumen. If carotenoids are substrates of P-glycoprotein or other transporters like breast cancer resistance protein or multidrug resistance-associated proteins (BCRP) or multidrug resistance-associated proteins (MRPs), the inhibition of piperine would reduce this efflux, allowing more carotenoids to be absorbed.^25^

The proposed mechanisms are summarized in Table 1. Understanding these intricate mechanisms is crucial for optimizing dietary strategies and potentially developing targeted interventions to maximize carotenoid utilization.

**Table 1.** Postulated Mechanisms of Synergistic Action between Piperine and Dietary Lipids in Enhancing Carotenoid Bioavailability

Mechanism category	Specific action of piperine	Specific role of dietary lipids	Combined synergistic outcome for carotenoids	References
Enhanced the absorption of piperine	Modulate membrane dynamics and inhibit enzymes and transporters	Facilitate the emulsification and micellar solubilization of carotenoids and piperine	Enhances the absorption of piperine and positions it at the absorption site	Sharma et al (2024),^4^ Chopra et al (2016)^33^
Enhanced micellar phase and stability	Potentially alters micelle properties; antioxidant protection within micelle/lumen	Essential for carotenoid solubilization and micelle formation	Improved carotenoid stability and delivery to enterocyte	Sharma et al (2024),^4^ Johnson et al (2022),^6^ Kopec et al (2017)^35^
Increased membrane permeability	Alters lipid milieu of enterocyte membrane, increasing fluidity/permeability	Deliver carotenoids via micelles to the altered membrane surface	Facilitated passive diffusion/partitioning of carotenoids into enterocytes	Khajuria et al (1998)^27^
Modulation of intestinal transporters	Inhibits P-glycoprotein (efflux pump); potential effects on uptake transporters	Present carotenoids as substrates for transporters	Reduced efflux of absorbed carotenoids; potentially enhanced uptake of carotenoids	Nguyen et al (2021)^25^
Inhibition of hepatic metabolism	Inhibits hepatic CYP3A4 and glucuronidation enzymes	Facilitate chylomicron remnant delivery of carotenoids to the liver	Reduced first-pass hepatic clearance of carotenoids, leading to higher systemic levels	Bhardwaj et al (2002),^10^ Atal et al (1985),^23^ Bi et al (2019)^24^

## CONCLUSION AND FUTURE PERSPECTIVES

The bioavailability of dietary carotenoids is a complex process, often limited by factors inherent to the carotenoids themselves, the food matrix, and host physiology.^4^ Dietary lipids play an essential role in facilitating carotenoid absorption, by aiding their release, solubilization into micelles, and subsequent transport into enterocytes. Emerging and historical evidence indicates that piperine, the principal bioactive alkaloid in black pepper, acts as a potent bioenhancer through multiple mechanisms, including the inhibition of metabolic enzymes and efflux transporters, and the modulation of intestinal membrane properties.^32^

This review examined the scientific basis for a synergistic interaction between dietary lipids and black pepper/piperine in enhancing carotenoid bioavailability. The current body of evidence, derived from mechanistic studies and a key human clinical trial (2022),^21^ strongly suggests that the co-consumption of these components can lead to a greater increase in carotenoid absorption and systemic availability than expected from their individual effects. This synergy was demonstrated for lutein, α-carotene, β-carotene, and lycopene,^21^ with piperine also reported to increase β-carotene’s serum response significantly.^36^ Mechanistically, this may involve piperine in black pepper increasing the absorption of intact carotenoids through actions like the inhibition of cytochrome P450s such as CYP3A4, and alteration of intestinal membrane permeability.^37^ At the same time, lipids provide the necessary vehicle for delivering carotenoids to this piperine-primed absorptive environment.

Despite these promising findings, the strength of the current evidence warrants further substantiation. More comprehensive, well-controlled human intervention studies are critically needed. These studies should explore a wider array of dietary carotenoids, investigate various food matrices, define optimal dose–response relationships for both piperine and dietary lipids, and assess long-term efficacy and safety. Elucidating the direct effects of piperine on specific carotenoid uptake transporters in humans remains a key area for mechanistic research.^11^

The potential nutritional and health benefits of optimizing carotenoid bioavailability through this natural, food-based approach are considerable. Harnessing the synergy between common dietary components such as lipids and a widely used spice like black pepper could offer a practical, cost-effective, and culturally acceptable strategy for improving carotenoid bioavailability. This may contribute to preventing vitamin A deficiency in vulnerable populations and reducing the risk of chronic diseases linked to oxidative stress.^4^ Moreover, using whole black pepper instead of isolated piperine may provide additional health benefits beyond enhancing bioavailability. This is because black pepper contains a variety of phytochemicals, such as essential oils, terpenes, and flavonoids, that possess antioxidant, antimicrobial, and anti-inflammatory properties.^38^^,^^39^

Future research could not only fill the existing scientific gaps but also focus on translating these findings into effective dietary guidelines, innovative functional food products, and impactful public health initiatives. The continued exploration of such dietary synergies holds significant promise for advancing human health and nutrition, underscoring the intricate and beneficial ways in which food components can interact within the biological system.

## Data Availability

The data underlying this article will be shared on reasonable request to the corresponding author.
